# 

**Published:** 1862-03

**Authors:** 


### CONTENTS.

ORIGINAL ESSAYS AND COMMUNICATIONS.

The Wants of the Profession. By J. Taft, ........ 101
The Deciduous Teeth. By J. Taft, ........ 107

PROCEEDINGS OF SOCIETIES.

Minutes of the Tenth Annual Meeting of the Ohio Dental College Association, ........ 114

SELECTIONS.

Facial Neuralgia, ........ 116
Dr. Ware on General Theurapeutics, ........ 124
Therapeutical Application, ........ 131
The Value of Statistical Information to the Dentist, ........ 135
On the Intermaxillary Bone and its Relations to Hare-lip and Cleft Palate, ........ 137

CORRESPONDENCE.

Letter from Philadelphia, ........ 140

EDITORIAL.

Atmospheric Pressure Plates, ........ 142
Cincinnati Dental Association, ........ 144
Peccavi, ........ 144
Rubber Attachment, ........ 146
A Proposition, ........ 147

